# Supplementary material for: Decline in seasonal predictability potentially destabilized Classic Maya societies
Source: Commun Earth Environ. 2023 Mar 17;4(1):82. doi: 10.1038/s43247-023-00717-5 (PMC11041697; doi:10.1038/s43247-023-00717-5)
Supplement: Supplementary file 1 — Supplementary Information [file 43247_2023_717_MOESM1_ESM.pdf]

Decline in seasonal predictability potentially destabilized Classic Maya societies: Supplementary Information

Tobias Braun,[1,*] Sebastian F. M. Breitenbach,[2] Vanessa Skiba,[1] Franziska A. Lechleitner,[3] Erin E. Ray,[4] Lisa M. Baldini,[5] Victor J. Polyak,[6] James U. L. Baldini,[7] Douglas J. Kennett,[8] Keith M. Prufer,[4] and Norbert Marwan[1]

[1] *Potsdam Institute for Climate Impact Research (PIK)*
Member of the Leibniz Association
14473 Potsdam, Germany

[2] *Department of Geography and Environmental Sciences*
Northumbria University
Newcastle upon Tyne, NE1 8ST, UK

[3] *Department of Chemistry, Biochemistry and Pharmaceutical Sciences*
and Oeschger Centre for Climate Change Research
University of Bern
3012 Bern, Switzerland

[4] *Department of Anthropology*
University of New Mexico
87131 Albuquerque, USA

[5] *School of Health & Life Sciences*
Teesside University
TS1 3BX Middlesbrough, UK

[6] *Radiogenic Isotope Laboratory*
Earth and Planetary Sciences
University of New Mexico
87131 Albuquerque, USA

[7] *Department of Earth Sciences*
Durham University
DH1 3LE Durham, UK

[8] *Department of Anthropology*
University of California
CA 93106 Santa Barbara, USA

[*] tobraun@pik-potsdam.de

I. SUPPLEMENTARY DISCUSSION

The age model of the studied record is shown in [1]. We use the 2σ age uncertainty from the distribution of COPRA modelled ages given at each sampled depth as an estimate for the 95% confidence bounds (Fig. S2). Sampling resolution and long-term evolution of stable isotope records and trace element records (Ba/Ca, Sr/Ca, U/Ca) is shown in Fig. S3. Prior to spectral analysis, the computation of proxy correlations and the recurrence analysis, we apply a detrending based on Singular Spectrum analysis (SSA) [2] (Fig. S4.). Using SSA, a non-stationary time series can be decomposed into a sum of components that capture time scale-specific modes of variability, i.e., trends, periodic cycles and fast variations/noise. First, a trajectory matrix is generated from time-shifted copies of the time series by chosing a window length w. Next, a singular value decomposition of the trajectory matrix and grouping of eigenvalues yields the SSA components. We only use the first component to detrend the age model medians and most central proxy realizations. Multi-decadal correlations between δ^{13}C and δ^{18}O (as discussed in Fig. 4 of the main manuscript) are computed based on the residuals that result after extracting the first SSA component ($w \approx 35$yr) (Fig. S4 A/B). Both the spectral and recurrence analysis are based on the full ensemble of age model realizations from which each is individually detrended with the first SSA component ($w \approx 10$yr, (Fig. S4 C/D).

Figure SS5 allows a detailed inspection of variability at seasonal time scales as retrieved after detrending of both stable isotope record's most central realization (MCR, see methods in the main manuscript). Their correlation at seasonal time scales is continuously positive (and mostly significant tested against AR(1)-surrogates) throughout the Common Era (Fig. S6). At multi-decadal time scales, periods of insignificant and significantly negative proxy correlation stand out (Fig. S7).

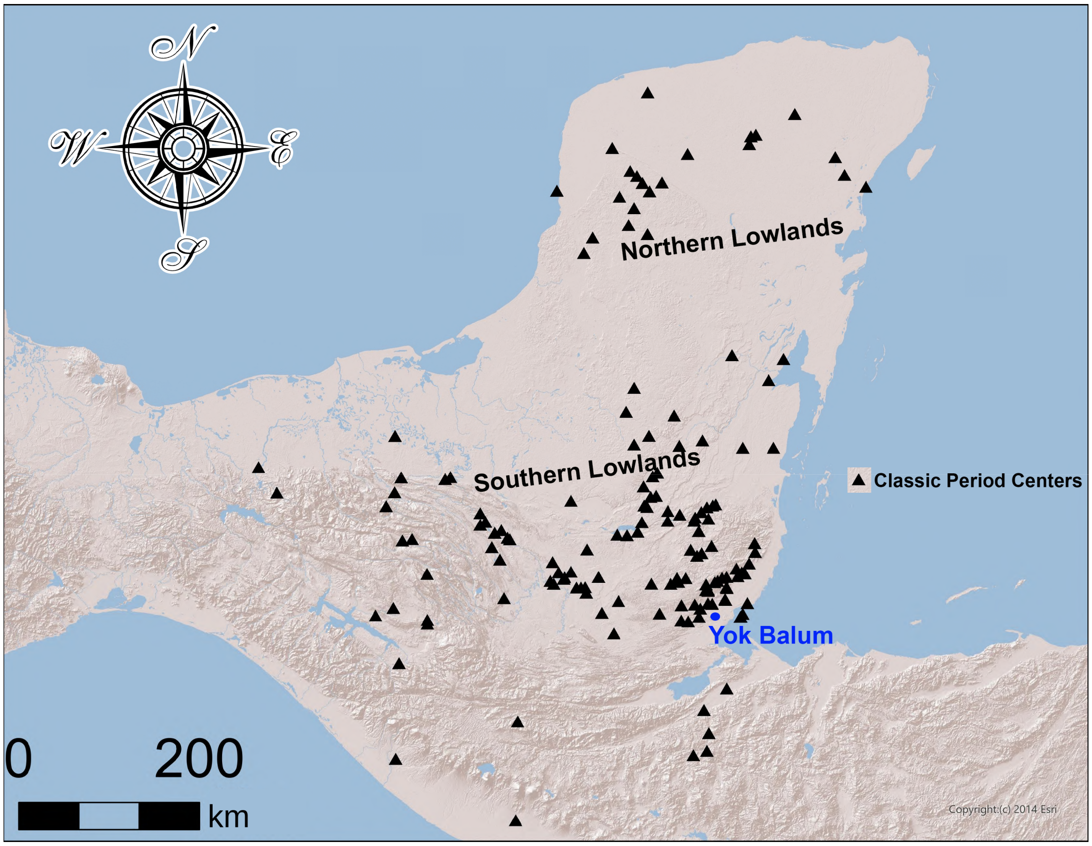

Fig. S1. Map of the Yucatán Peninsula, indicating the study site (Yok Balum) and Classic Period Centers in the Southern and Northern Maya Lowlands. (Map image is the intellectual property of Esri and is used herein under license. Copyright ©2020 Esri and its licensors. All rights reserved.)

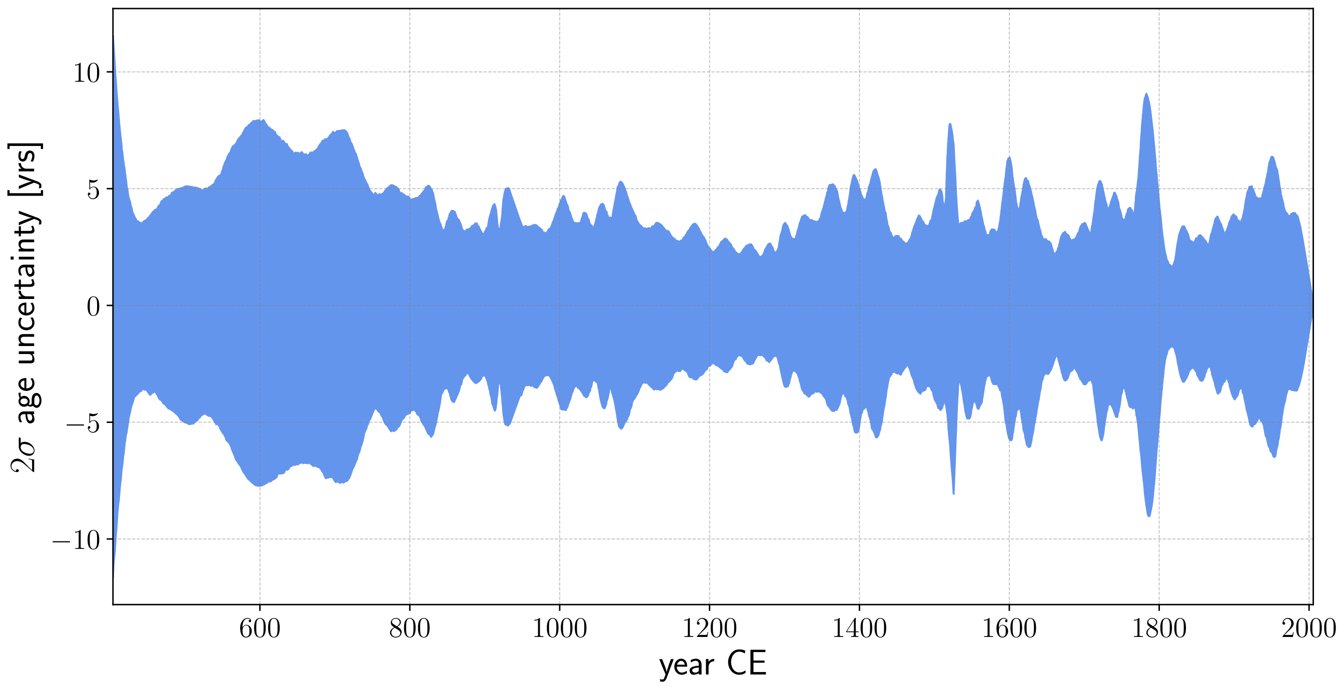

Fig. S2. Evolution of 2σ age uncertainty after application of COPRA age model (50 U/Th-dated depths).

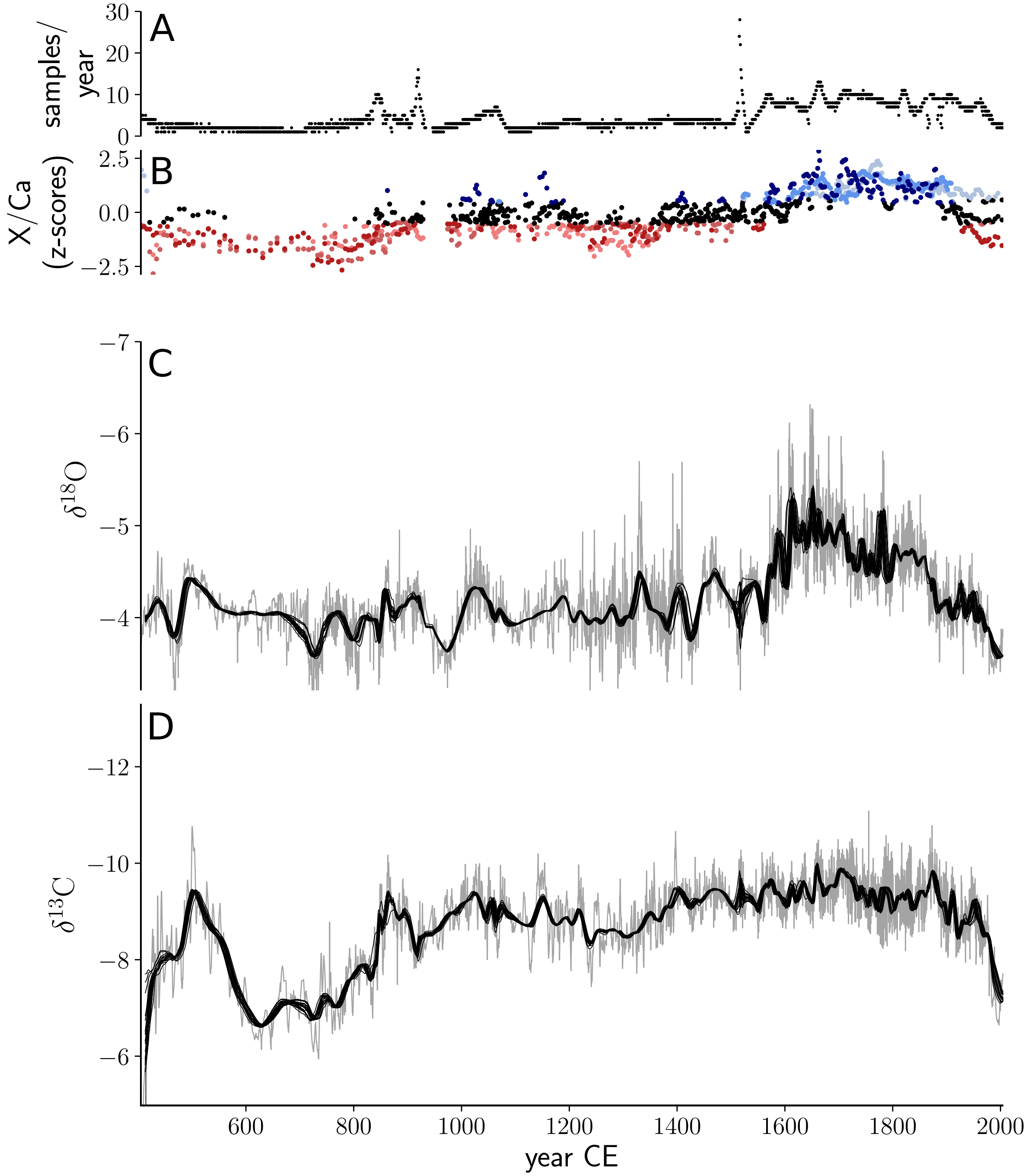

Fig. S3. Long-term variability of YOK-G proxy records. (**A**) Sampling resolution (samples/year) in the age-modelled stable isotope records. (**B**) z-scores of trace elements (Ba/Ca, Sr/Ca, U/Ca) to substantiate interpretation of stable isotope records. δ^{18}O (**C**) and δ^{13}C (**D**) long-term trends are individually estimated for all δ^{18}O and δ^{13}C proxy realizations as the first component of a Singular Spectrum Analysis ($w \approx$ 10yr, trends for 50 realizations displayed). The maximum-correlated age model realizations for both isotope records (gray) additionally exhibit fluctuations at seasonal time scales.

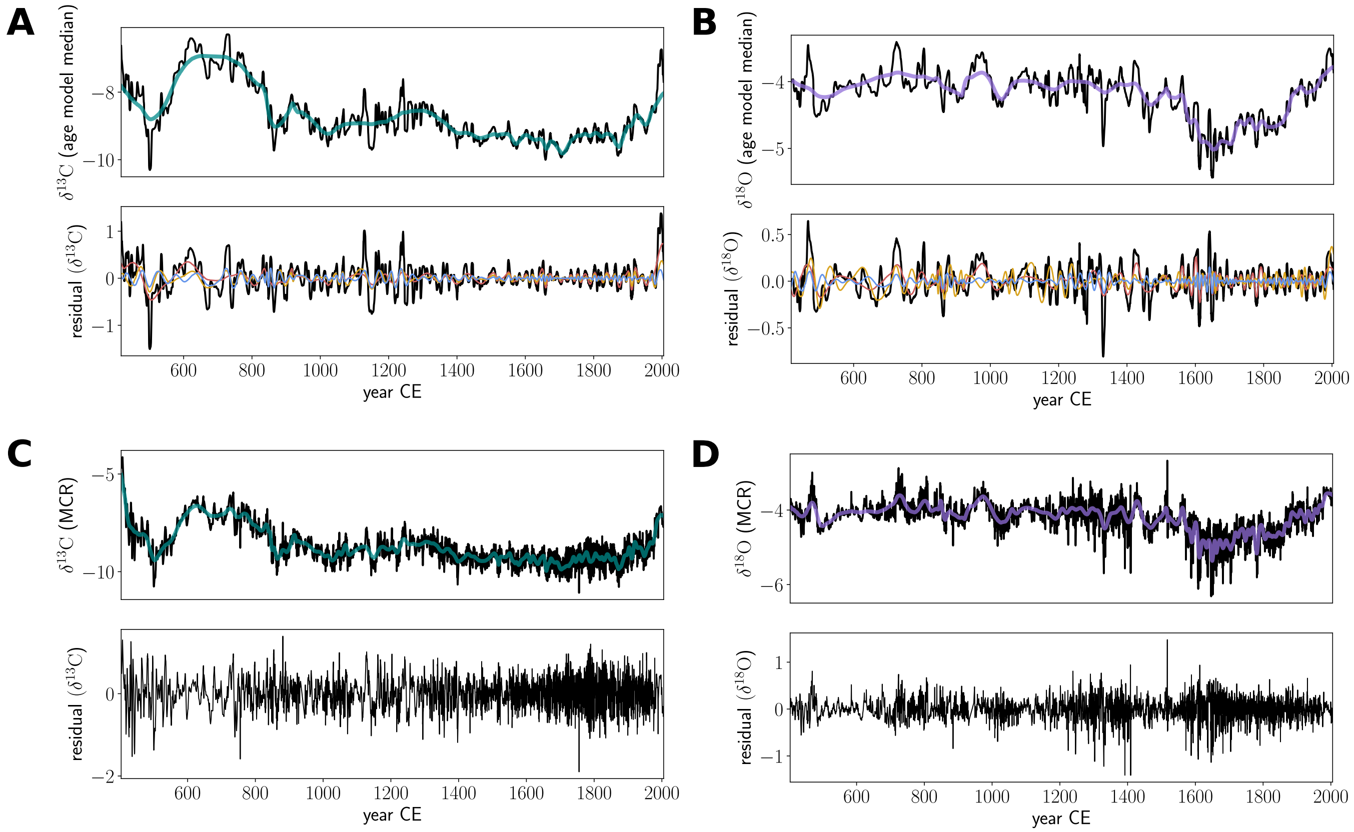

Fig. S4. Detrending of age model medians and most central realizations using Singular Spectrum analysis (SSA). (**A**) Detrending of δ^{13}C age model median with the first component returned by SSA (cyan). The residual is shown below, including the next three leading SSA components (red, yellow, blue). (**B**) same as (A), but for δ^{18}O. (**C**) Detrending of δ^{13}C most central realization with the first component returned by SSA (cyan). The residual is shown below, no additional components displayed for the sake of visibility. (**D**) same as (C), but for δ^{18}O.

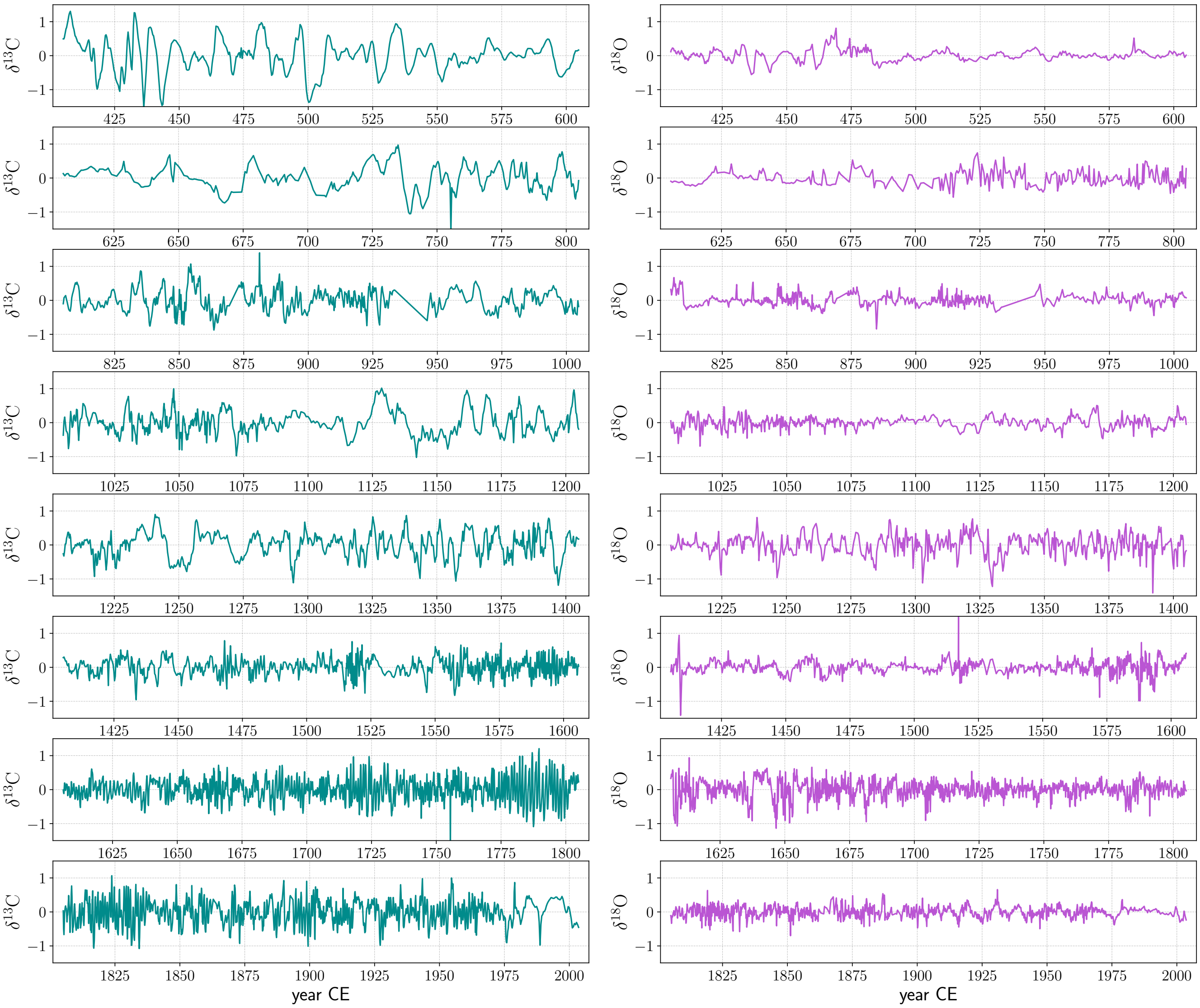

Fig. S5. Most central realizations of δ^{13}C (left) and δ^{18}O (right) for the full covered time period (after detrending). They act as a representation for the full ensemble of age model realizations as they have the highest average correlation to all other realizations.

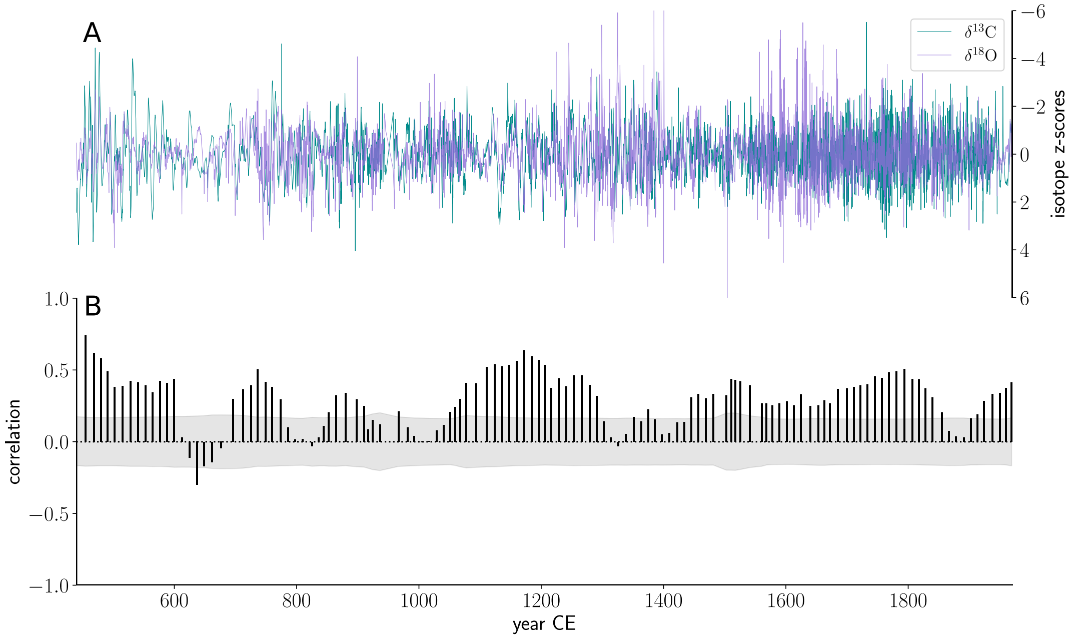

Fig. S6. (**A**) Most central realization z-scores of δ^{13}C and δ^{18}O after extraction of individual trends using SSA. (**B**) Averaged seasonal-scale correlation between 2000 pairs of δ^{13}C and δ^{18}O age model realization z-scores. Correlations are computed on 50 year-sliding windows and tested against 1000 (irregularly sampled) AR(1)-surrogates (gray shading).

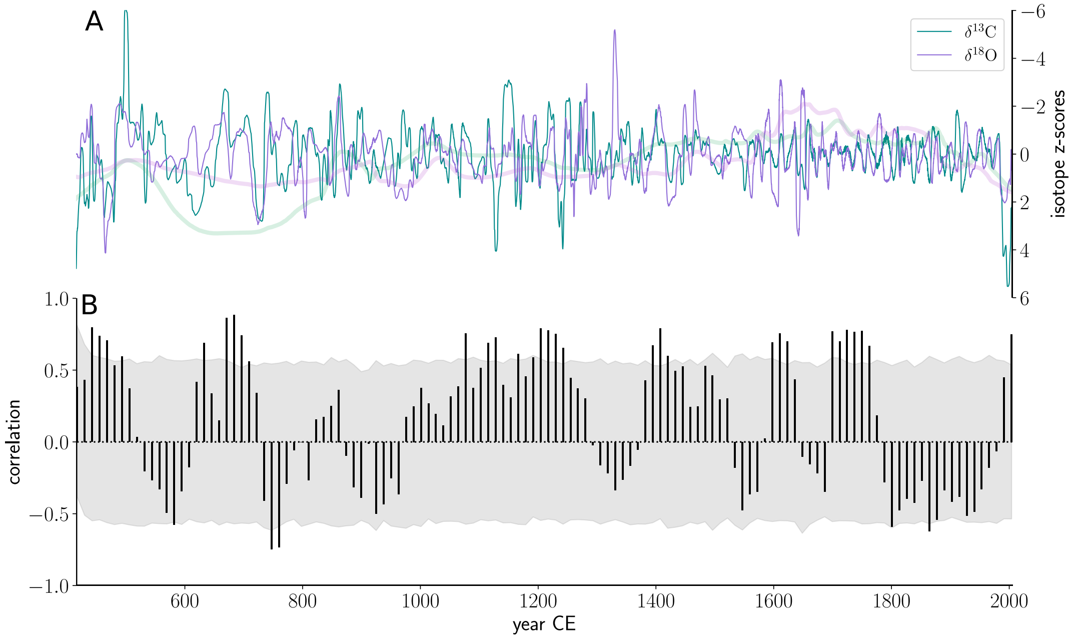

Fig. S7. (**A**) Age model median δ^{13}C and δ^{18}O z-scores after extraction of trends (shaded) using SSA. (**B**) Multidecadal correlations are computed on 50 year-sliding windows and tested against 1000 (irregularly sampled) AR(1)-surrogates (gray shading) (**B**).

II. SUPPLEMENTARY METHODS

We use Lomb-Scargle (LS) periodograms along with Continuous Wavelet Spectra (CWS) to examine the significance of the seasonal cycle and ENSO–multi-decadal-scale cycles throughout the Common Era for both stable isotope records. Both proxy records are split into five segments (430 - 650 CE, 650 - 950CE, 950 - 1400 CE, 1400 - 1800 CE and 1800 - 2005 CE) and Lomb–Scargle periodograms are computed for each segment separately. Welch spectra are computed to obtain robust estimates of spectral power with reduced noise intensity. We choose the number of sub-segments n_{seg} for each segment such that each sub-segment approximately covers 500 samples, corresponding to approximately $60 - 300\,\mathrm{yrs}$. Each sub-segment is weighted with a Blackman-window to limit the effect of spectral leakage. We choose twice the average Nyquist frequency $2f_{\mathrm{Nyq}} = 1/\Delta\bar{t}$ as an upper limit for the frequency range. Each frequency is spaced with $\Delta f = (n_{\mathrm{seg}} + 1)/2\sigma T \Delta\bar{t}$ whereas T is the length of a time series segment, $\Delta\bar{t}$ is the average sampling interval and $\sigma = 2$ is the oversampling factor. We repeat the computation of LS-periodograms for a given time period for each MC-realization and averaged afterwards. Significance of spectral peaks is assessed by means of AR(1)-surrogates [3]. AR(1)-surrogates are frequently used for hypothesis testing in the paleoclimate literature [4]. The underlying idea is that in a low-order approximation, proxy time series exhibit deterministic (e.g. seasonal) variability superimposed on a background of correlated noise. By sampling N_{s} surrogate time series that resemble the correlated noise, it is tested whether the observed value for a statistic can occur just 'by chance' or if its occurrence is significantly different from the modelled noise time series. It is important to note that all AR(1)-surrogates are generated on the real unevenly spaced time axis and thus also recover the features which are affected by sampling biases. An α-confidence level is obtained for each MC-realization separately and subsequently averaged.

For the computation of CWS for both stable isotope time series, we account for age-uncertainty following the scheme described in the methods to test whether the results obtained in [1] can be confirmed when uncertainties are included. In a first step, linear interpolation is applied to obtain a regular time axis with even spacing. The choice of the sampling interval Δt requires caution; if it is chosen too high, spurious serial dependence is imposed on the time series and will result in erroneous spectral peaks. In an attempt to limit the impact of spurious serial dependence, the root-mean-square error (RMSE) between the real LS-periodograms and LS-periodograms computed from interpolated time series is minimized. This optimization is displayed in Fig. S8 and yields a value of $\Delta t = 0.30$, averaged over the distinct optimal values obtained for δ^{13}C and δ^{18}O. This sampling interval is still low enough to assess seasonal variability ($\Delta t < 0.50$). Continuous Wavelet transformation (as described in [5]) is carried out using the PyCWT package in Python with a Morlet mother Wavelet. Significance of spectral power is tested for each realization by generating 100 irregularly sampled AR(1)-surrogates, applying the same interpolation procedure to each and computing the resulting 90%-confidence level. The respective 90%-quantiles are computed for each time instance since variations in the sampling density entail variations in the significance of spectral peaks. A 'MC-Wavelet spectrum' is obtained by counting the number of realizations which indicate significant spectral power for a given period and time instance. If more than half of all realizations indicate significant spectral power, the corresponding cycle at a given time instance is considered significant within the given age uncertainty.

The resulting MC-Wavelet spectrum (Fig. S12) shows less spectral background noise than a regular Wavelet spectrum (see Fig. S9/SS10 for comparison). Throughout some episodes, both proxy time series are not sufficiently resolved to reliably detect an annual cycle (see Fig. S3A, e.g. 700 CE) as the sampling frequency drops below the Nyquist frequency. It is well known that in such cases, aliasing can occur and may introduce spurious lower frequency cycles in spectral estimates. We perform a basic MC-sampling test to identify which aliased cycles might occur just due to a masked annual cycle. To this extent, a sinusoidal with annual periodicity is generated on the original irregular time axis and linear interpolation is applied as described above. Age uncertainty is mimicked by generating 2000 sinusoidals with random phases which are drawn from a $\mathcal{N}(0, \sigma_{\mathrm{a}})$-distribution with the real standard deviation of ages σ_{a} obtained from COPRA. An example of the interpolated and non-interpolated sinusoidal time series is shown in Fig. S11 for a time period where aliasing effects can be observed. Ten MC-Wavelets are computed (with different random seeds for the phase distribution) and averaged, resulting in a single MC-Wavelet spectrum for the sinusoidals. The white hatching in Fig. S12B/D indicates regions of masked seasonal variability caused by aliasing. Thus, any overlap between significant spectral power and the hatched spectral aliasing patches could actually reflect an annual cycle that can not be resolved rather than the indicated period.

Figure SS9/SS10 shows Wavelet spectra of the MCR of both stable isotope records. Comparing them to the MC-Wavelet spectra shown in Fig. S12B/D, it becomes clear that the latter recover most of the significant cycles that are found for the MCR, demonstrating that these cycles are significant also when age uncertainty is taken into account. This adds confidence to the findings from the LS-spectra and suggests that especially after 1400 CE, the seasonal cycle remains relatively stable. These observations align with the results in [1] where the same δ^{13}C record was studied but without computing MC-Wavelet spectra based on single proxy realizations. The finding of a strengthened, more pronounced seasonal cycle in the post-1400 CE period is further bolstered by the MC-Wavelet spectrum of all δ^{18}O realizations in Fig. S12D where a high number of realizations indicate relatively high power in the annual band. These

results complement and corroborate the transition in rainfall seasonality detected in the YOK-G δ^{13}C record [1].

Between 435-950 CE, several years have less than two samples and do consequently not allow for reliable extraction of a seasonal cycle ($f < f_{\mathrm{Nyq}} = 2$/year). Some overlap of real spectral power and white hatched regions hints at the possibility that during these periods, a muted seasonal cycle could have also been present but can not be extracted realiably due too the low growth rate of the stalagmite during persistent drought conditions.

Beyond the observed variations in seasonal variability, distinctiveness of inter-annual and (multi-)decadal-scale cycles are also found to vary throughout the records. Both proxy records suggest that ENSO- (2-8 years) and (multi-)decadal-scale variability were more pronounced in the pre-1400 CE period. The presence of ENSO-scale variability is particularly strong in the δ^{13}C record.

We show the LS spectra after averaging single-realization based spectra over all 2000 (detrended) realizations of δ^{13}C and δ^{18}O for the five time intervals in Fig. S12A/C. The (cyan and purple) triangles yield a less conservative estimate of significant cycles without accounting for age uncertainty and indicate that for the MCR, a seasonal cycle can be identified for each time interval and both proxies with 95% confidence. Yet, only between 950-2005 CE a robust seasonal cycle is identified in the δ^{13}C record when accounting for age uncertainty (cyan shading).

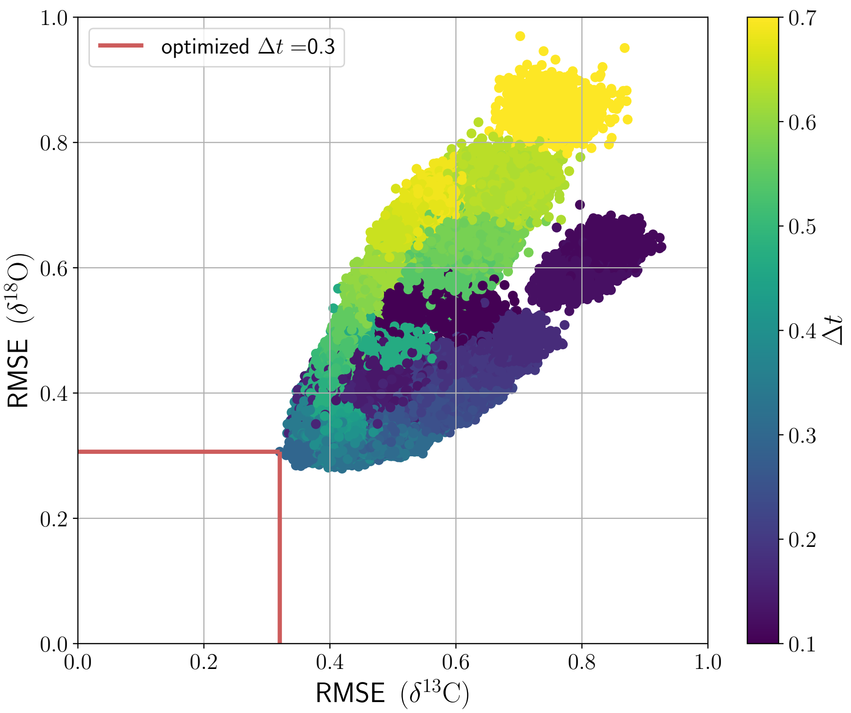

Fig. S8. Optimization of sampling interval Δt for linear interpolation (used exclusively in Wavelet analysis). Root-mean-square errors (RMSE) between Lomb-Scargle spectra for interpolated and non-interpolated time series are shown for all realizations of both stable isotope records. The trade-off value of $\Delta t = 0.30$ years for which the sum of both RMSEs is minimized is indicated in red.

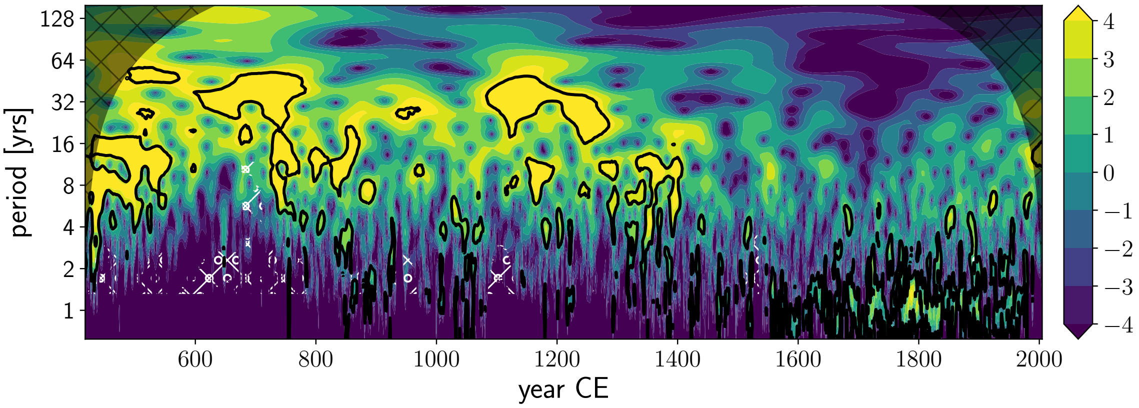

Fig. S9. Continuous wavelet spectra for most central realizations of δ^{13}C age model ensembles. Spectral power is tested against a red noise background spectrum, generated from 2000 irregularly sampled and interpolated AR(1)-surrogates (90% confidence level).

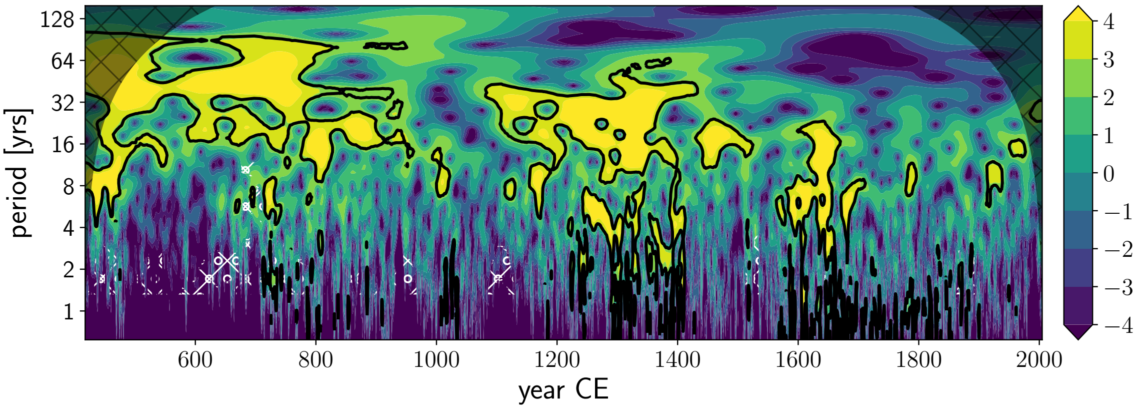

Fig. S10. Continuous wavelet spectra for most central realizations of δ^{18}O age model ensembles. Spectral power is tested against a red noise background spectrum, generated from 2000 irregularly sampled and interpolated AR(1)-surrogates (90% confidence level).

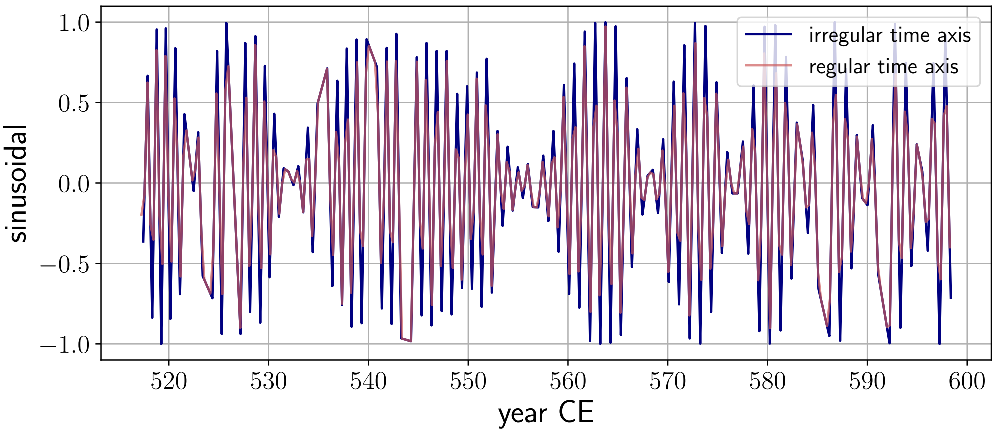

Fig. S11. Illustration of aliasing effect that occurs when sampling resolution falls below the Nyquist frequency of 2 samples/year based on a synthetic sinusoidal time series with a period of one year and the real proxy time axis (blue). Higher periods are appearing as an artefact due low sampling resolution and are not eliminated by linear interpolation (red). 1000 sinusoidals with random phases are generated to inform about the resulting spurious but significant longer cycles in the Wavelet spectrum (suppl. fig. 8).

The recurrence analysis described in the methods of the main manuscript results in a total of 69 recurrence matrices on sliding windows for each proxy-realization. Six exemplary recurrence plots are shown in Fig. S13 for both stable isotope records. In the recurrence quantification analysis, the mean diagonal line length was used to describe changes in the predictability time of hydroclimate variability. In some of the examples (e.g. third row from top), striking block structures can be seen that solely arise from a finite-sampling effect of the (m)edit-distance discussed in [6]. Thus, sampling-rate constrained (SRC)-surrogates that reproduce this spurious effect and mimic the resulting recurrence properties are generated (displayed in the right columns). While the general structure (including the macroscopic block structures) are conserved by the surrogates, the microstructures (e.g. diagonal lines) differ between the real and the surrogate-based RPs.

In Fig. 2-4 of the main manuscript, the mean diagonal line length (denoted by T_{pred}) is corrected for this effect based on the mean diagonal line lengths $T_{\text{pred}}^{(\text{surr})}$ of the surrogate RPs for each window, yielding τ_{pred}. We conduct the same analysis for the δ^{18}O record (Fig. S14B/D). The mean predictability time τ_{pred} computed from δ^{18}O declines more rapidly but supports low seasonal rainfall predictability during the CPC. After an increase towards 1200 CE that aligns well with the mean predictability time suggested by δ^{13}C, seasonal rainfall amount appears well predictable during the LIA around 1600 CE (Fig. S14B). Both stable isotope records indicate declining seasonal predictability towards the modern period. Linking seasonal predictability to the hydroclimatic background state, both stable isotope records show significantly different relationships between seasonal predictability and long-term isotopic averages than expected from the random null model (gray dots, Fig. S14C/D). Furthermore, both stable isotope records showcase that a simple linear relationship in the sense ‘the drier, the less predictable’ would not suffice to capture the complexity of how seasonality and long term aridity are intertwined in the Maya lowlands.

The driest interval in the δ^{13}C record coincides with a pronounced decline in predictability. Here, two factors converge that affect the reconstruction. On the one hand, intensifying droughts are likely expressed as lengthened and possibly intensified dry seasons along with a decline in wet season length and wet season rainfall amount. This reduction in effective moisture supply would potentially go hand in hand with a less predictable timing (i.e., onset and withdrawal) of the rainy season. Under drought conditions, the tropical rainband (ITCZ) would not develop a well-organised, pan-regional deep convection, but be characterised by more individual and erratic thunderstorms, both spatially and temporally. Such atmospheric conditions would prove more difficult to predict. On the other hand, ensuing drought conditions – if severe and long enough – would diminish the epikarst water reservoir such that dripwater supply to the stalagmite would dwindle, eventually resulting in diminished carbonate precipitation. This means that, if subsampling resolution remains fixed (as in our case), the temporal sampling resolution diminishes (i.e., a given sample would integrate more time during drought compared to periods with faster stalagmite growth). At some point, this would impact τ_{pred} as the given samples are not probing the true dispersion of the signal. While the surrogate-based method from [6] enables us to compare such years with years of high resolution, integration biases beyond this cannot be assessed.

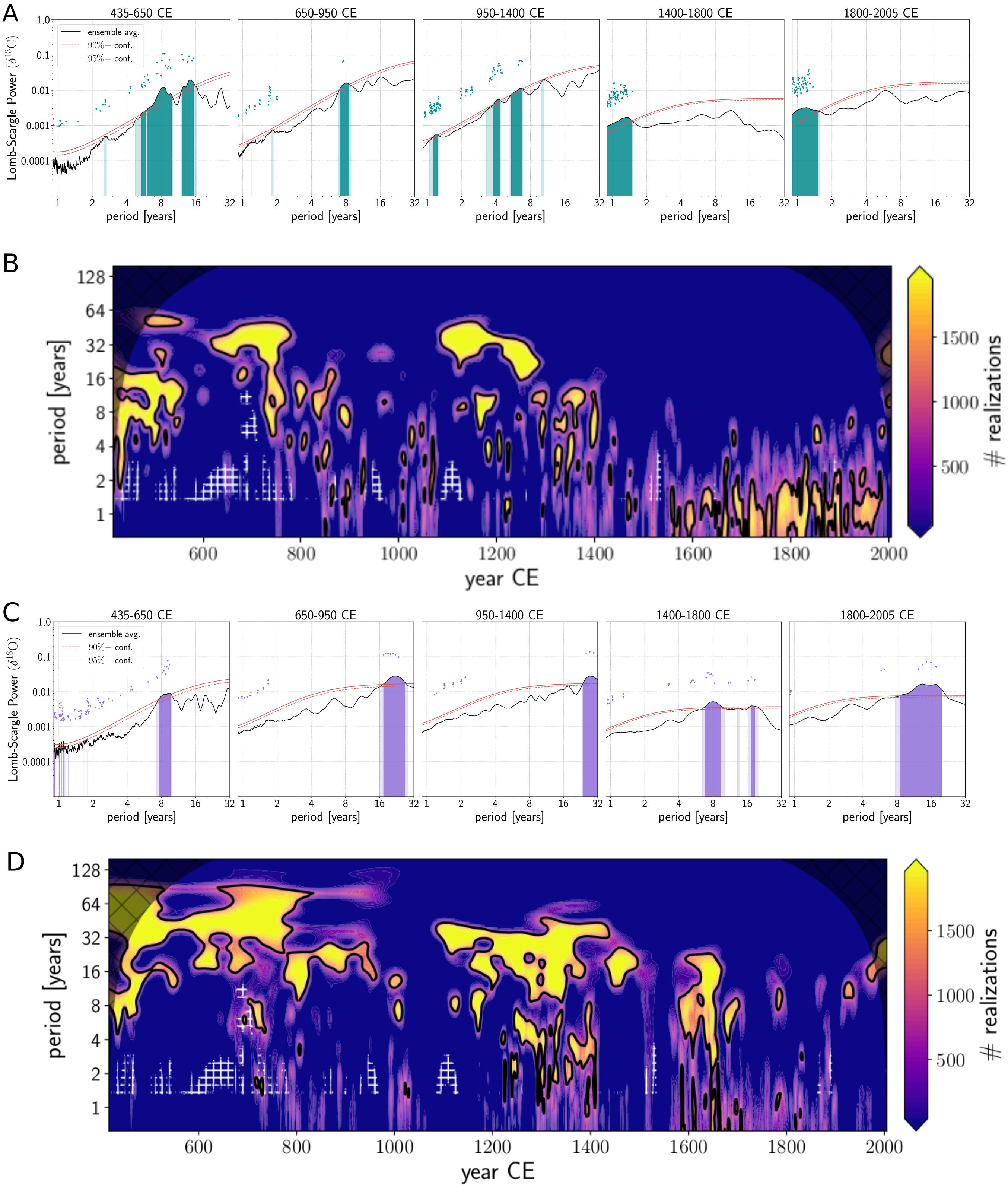

Fig. S12. Time-frequency analysis of stable isotope records: LS spectra for five segments of the δ^{13}C (**A**) and δ^{18}O (**C**) record respectively. Median spectral power, computed from 2000 detrended proxy realizations (black) is tested for significance using irregularly sampled AR(1)-surrogates (red). Significant cycles in the MCR are indicated by triangles. Computation of an individual continuous wavelet spectrum for each (linear interpolated & detrended) proxy realization results in a single MC-wavelet spectrum, shown for δ^{13}C (**B**) and δ^{18}O (**D**) respectively. Color coding denotes the number of proxy realizations that indicate a significant cycle. Black contours mark cycles for which more than half of all realizations indicate significant spectral pwoer. White hatching marks regions of potential aliasing.

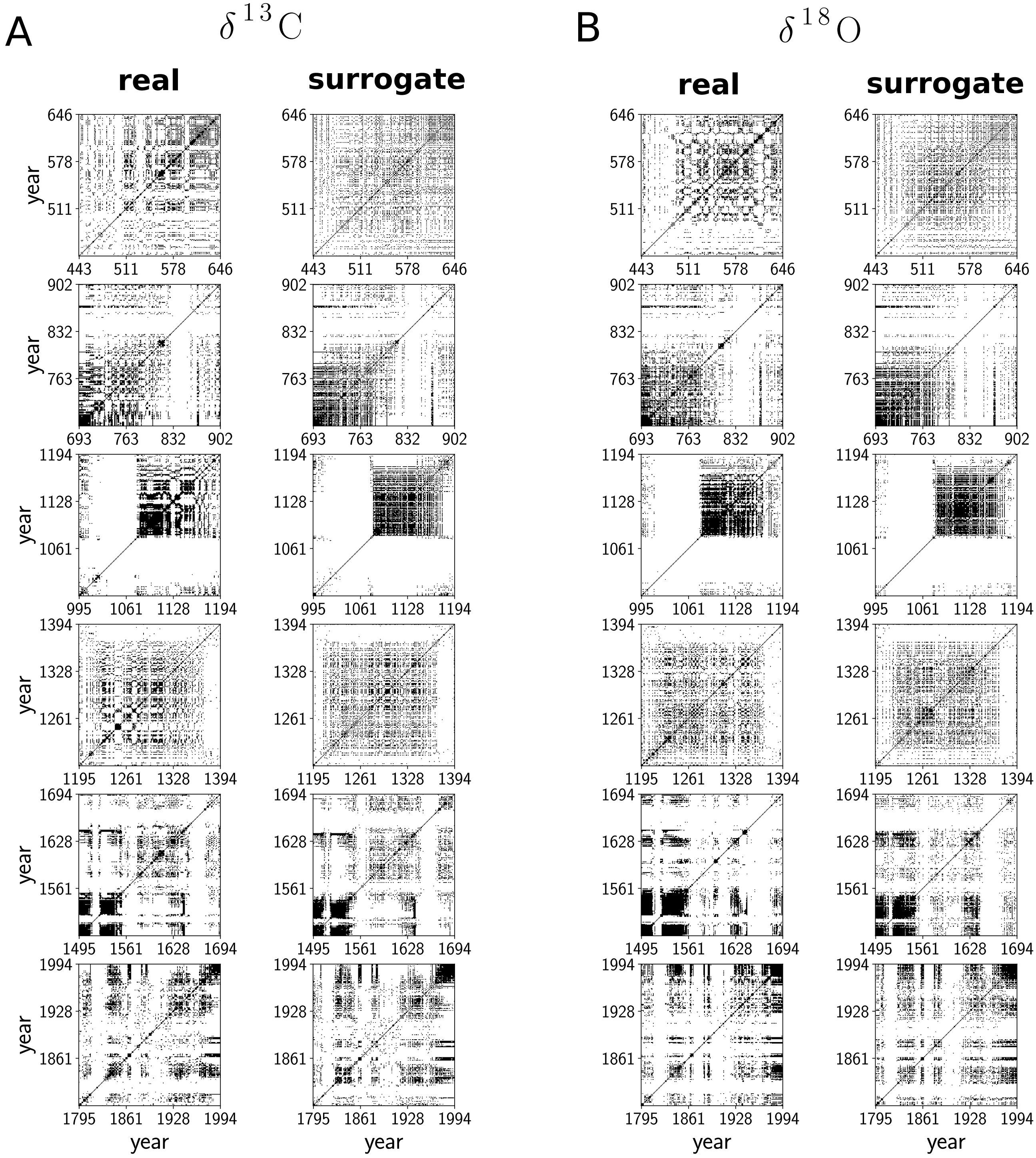

Fig. S13. Exemplary recurrence plots from the sliding window recurrence analysis (**A**: δ^{13}C, **B**: δ^{18}O). Real RPs are shown in the left column and SRC-surrogate RPs are shown in the right column, respectively. Each RP covers a time window that correspond to 200 years and is computed with the (m)edit distance method.

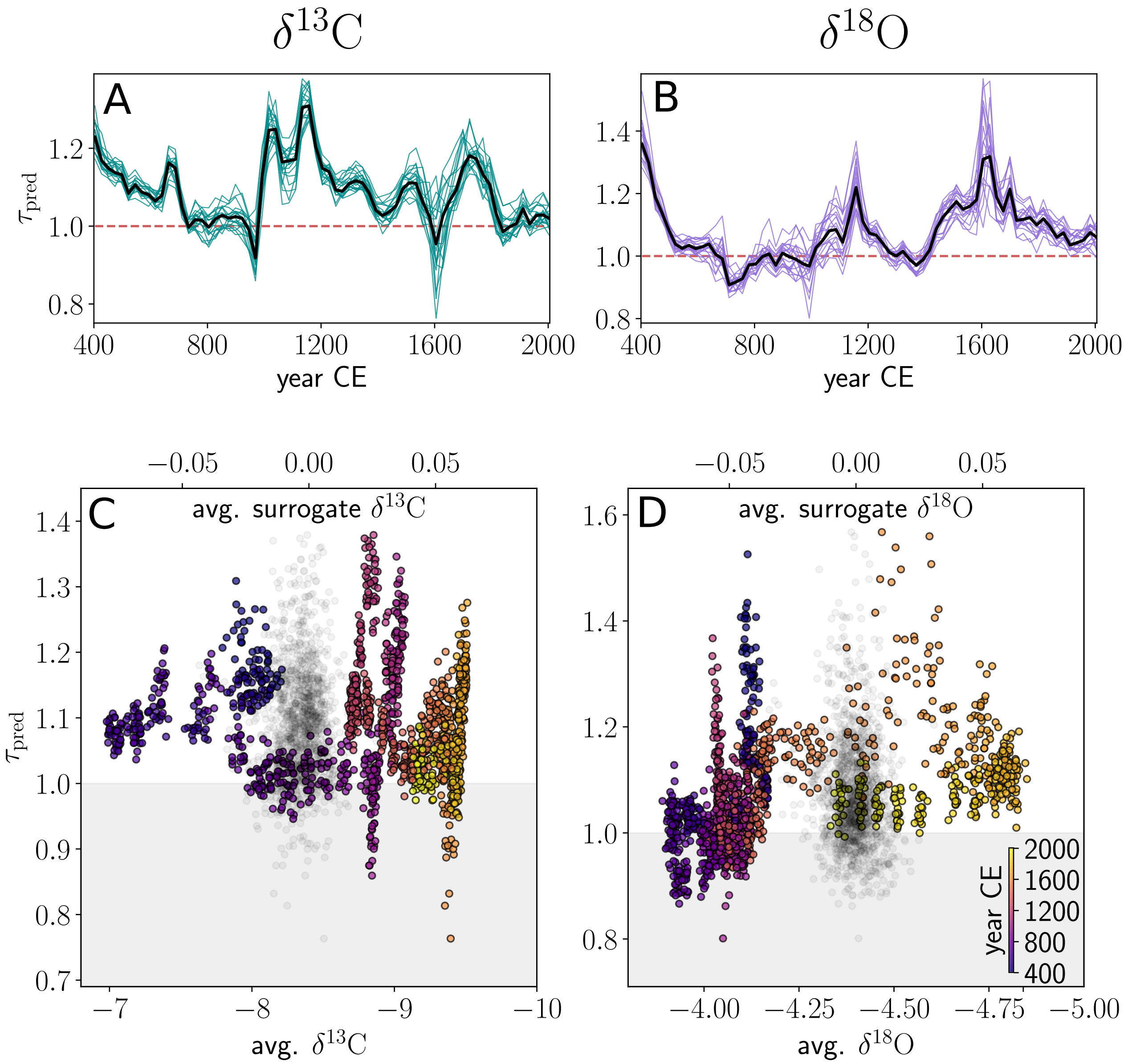

Fig. S14. (**A**/**B**) Predictability of sub-annual rainfall distribution, given by relative mean predictability times τ_{pred} of 20 detrended proxy realizations for each isotope. $\tau_{\text{pred}}(t)$ with reference value of $\tau_{\text{pred}}^{(\text{ref})} = 1$ (red dashed line) is displayed for δ^{13}C (cyan) and δ^{18}O (purple) to display transitions in time. (**C**/**D**) Scatter plots show relation between dry/wet episodes to predictability of the sub-annual rainfall distribution. Values on the x-axis indicate general hydroclimatic conditions during the respective period (average of non-detrended isotope values) while τ_{pred} is computed from detrended proxy realizations. Gray shading(/dashed red line) indicates predictability times $\tau_{\text{pred}} < 1$ that are not larger than expected from a random proxy-surrogate with the same sampling resolution. The dependency structure that results from the null model is indicated by gray dots.

In the manuscript, we describe a scenario for which seasonal variations δ^{13}C could be muted and hamper predictability (scenario (3)). This could result from two different sources: firstly, the reduction in seasonality could be genuine, i.e. wet season is muted which would shrink seasonal amplitude compared to 'normal' years whilst sampling resolution remains sufficiently high to resolve such changes. Secondly, the potential genuine reduction in seasonality could be overprinted by sampling effects, i.e. a reduction in sampling resolution that artificially integrates δ^{13}C values and, in turn, would also shrink seasonal amplitude. We design a toy model to obtain an estimate of how strongly the second

scenario could affect seasonal predictability τ_{pred} during the CPC (Fig. S15). To this extent, we generate regularly sampled sinusoidals with a temporal resolution that is equivalent to the year with highest resolution of YOK-G δ^{13}C from 403-1000 CE (14 samples/year). Next, we downsample each sinusoidal to the real records resolution. We pursue two downsampling strategies that reflect distinct possible scenarios that are conceivable during dry conditions: in the *constant growth* scenario, we assume stalagmite growth was low but steady all year and δ^{13}C measurements are integrated values. Consequently, we randomly pick n out of $N = 14$ samples with equal probability for each time of the year. For each year, the sinusoidal is downsampled to these n samples by integration of all values in between, i.e. averaging between pairs of time instances (t_i, t_{i+1}), $i = 1, \dots, n-1$. In the *seasonal bias* scenario, we assume that drips only occur in the wet season such that the measured δ^{13}C samples are predominately taken from the wet season. This is implemented by drawing samples from the sinusoidal with a probability that is biased towards the wet season.

We define (normalized) probabilities for each of the 14 time points such that these are proportional to the annual rainfall profile from the study region, i.e. following a sinusoidal that peaks in July/August for each year. The n randomly drawn samples are kept and all others are discarded (no integration is performed). Segments from two respective MC-realizations show that both scenarios yield distinct signatures of how the seasonal signal is muted (Fig. S15A/B). As an estimator for the expected change in seasonal predictability, we compute τ_{pred} for 30 MC-realizations for each scenario (with 20 SRC-surrogates each). We compute the relative declines in τ_{pred} returned by these model realizations from 403 CE to 820 CE and 403 CE to 1000 CE (Fig. S15C) as these years yield local minima in the real predictability of δ^{13}C. The toy model indeed favors scenarios of declining predictability for both scenarios. Note that the model does not include any variations in predictability *per se* but only variations in sampling resolution. The observed decline in predictability, however, are considerably smaller than the real decline in predictability based on δ^{13}C for both time spans and both scenarios. Finally, we compute the strongest decline that is generated from the model for each realization, regardless of the corresponding time period and compare it to the observed decline in τ_{pred} from 403-1000CE. Even with this conservative estimator, the observed decline is still stronger, adding confidence to our main finding.

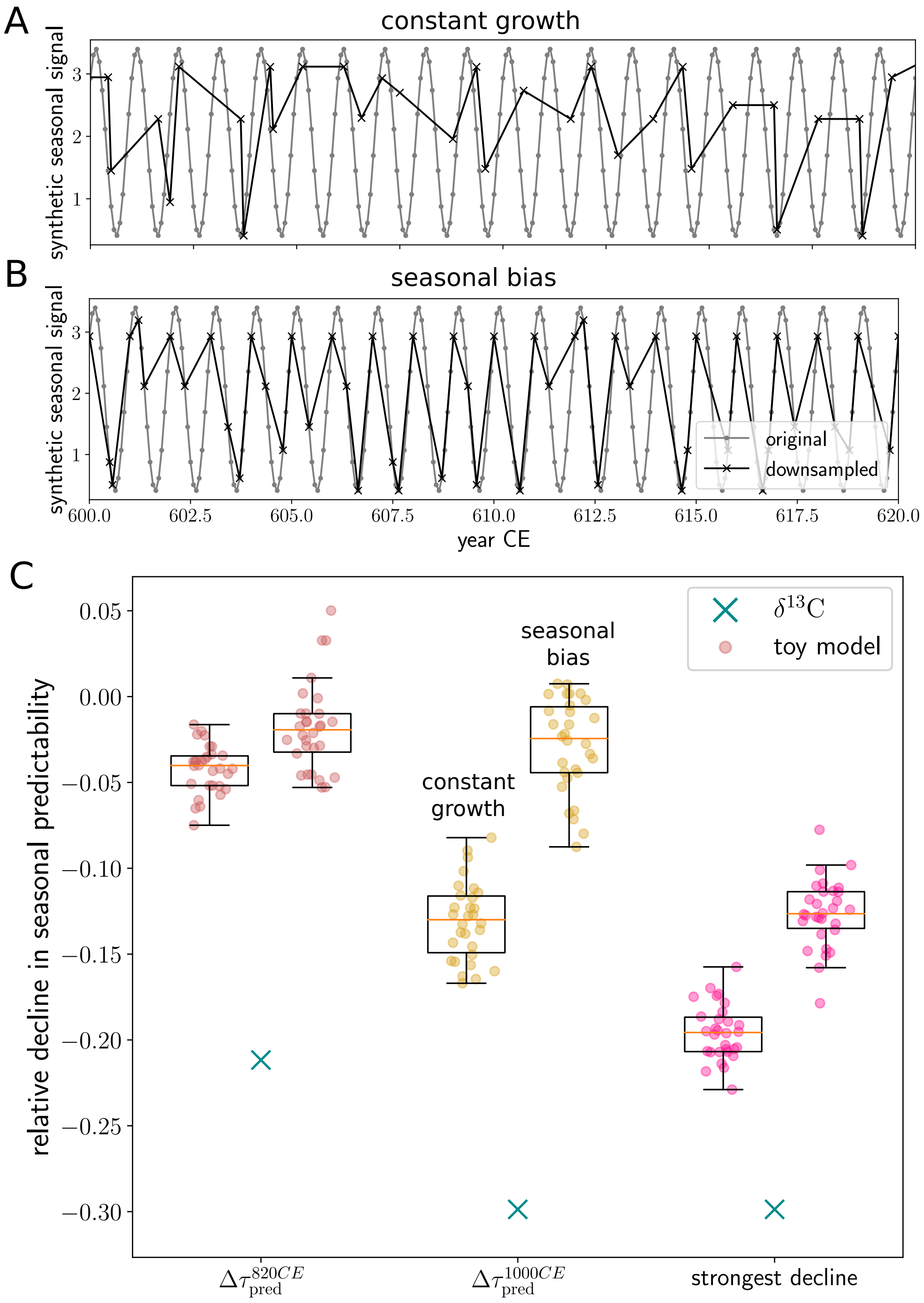

Fig. S15. Estimation of potentially biasing effects on seasonal predictability, using toy model simulations. In the *constant growth* scenario, stalagmite growth is assumed to be low but constant over the year and samples are integrated (A). In the *seasonal bias* scenario, the stalagmite is assumed to be fed by drips from the wet season, thus discarding dry season samples with highest probability (B). Relative declines in seasonal predictability as returned by the model for both scenarios and three different periods (boxplots) are compared to the real decline in τ_{pred} based on the YOK-G δ^{13}C record (crosses) (C).

SUPPLEMENTARY REFERENCES

[1] Y. Asmerom, J. U. L. Baldini, K. M. Prufer, V. J. Polyak, H. E. Ridley, V. V. Aquino, L. M. Baldini, S. F. M. Breitenbach, C. G. Macpherson, and D. J. Kennett, "Intertropical convergence zone variability in the Neotropics during the Common Era," Science Advances **6** (2020).

[2] R. Vautard and M. Ghil, "Singular spectrum analysis in nonlinear dynamics, with applications to paleoclimatic time series," Physica D: Nonlinear Phenomena **35**, 395–424 (1989).

[3] M. Mudelsee, "Tauest: A computer program for estimating persistence in unevenly spaced weather/climate time series," Computers & Geosciences **28**, 69–72 (2002).

[4] M. Schulz and M. Mudelsee, "Redfit: estimating red-noise spectra directly from unevenly spaced paleoclimatic time series," Computers & Geosciences **28**, 421–426 (2002).

[5] C. Torrence and G. P. Compo, "A practical guide to wavelet analysis," Bulletin of the American Meteorological Society **79**, 61–78 (1998).

[6] T. Braun, C. N. Fernandez, D. Eroglu, A. Hartland, S. F. M. Breitenbach, and N. Marwan, "Sampling rate-corrected analysis of irregularly sampled time series," Physical Review E **105**, 024206 (2022).
